# *Leptochilus dolichophyllus* (Polypodiaceae), a New Hemiepiphytic Species From Fujian, China

**DOI:** 10.1002/ece3.72293

**Published:** 2025-10-19

**Authors:** Hou‐Hua Fu, Cheng‐Yuan Zhou, Xiong‐De Tu, Liang Ma, Shi‐Pin Chen, Hong‐Jin Wei

**Affiliations:** ^1^ College of Forestry Fujian Agriculture and Forestry University Fuzhou China; ^2^ Key Laboratory of National Forestry and Grassland Administration for Orchid Conservation and Utilization at Fujian Agriculture and Forestry University Fuzhou China; ^3^ Fujian Health College Fuzhou Fujian China; ^4^ Eastern China Conservation Centre for Wild Endangered Plant Resources Shanghai Chenshan Botanical Garden Shanghai China

**Keywords:** climbing habit, hemiepiphytism, *Leptochilus leveillei*, phylogeny, southeastern China

## Abstract

A new species, *Leptochilus dolichophyllus* (Polypodiaceae), from central‐northern Fujian, southeastern China, is described and illustrated. It is morphologically similar to *Leptochilus leveillei* , but differs by its hemiepiphytic habit and longer frond. Molecular phylogenetic analysis based on three plastid regions (*rbcL*, *rps4* + *rps4*‐*trnS*, and *trnL* + *trnL‐trnF*) demonstrates that the new species represents a distinct and well‐supported clade. Both morphological and molecular data provide compelling evidence for the recognition of *L. dolichophyllus* as a new species. Furthermore, the complete plastid genome of this new species is reported.

## Graphical abstract

We have identified one new *Leptochilus* species based on morphological observation and molecular evidence, *Leptochilus dolichophyllus* H.H.Fu & H.J.Wei, from central‐northern Fujian, southeastern China. Additionally, to provide valuable molecular resources for further studies, we assembled and annotated the complete plastome of this new species.

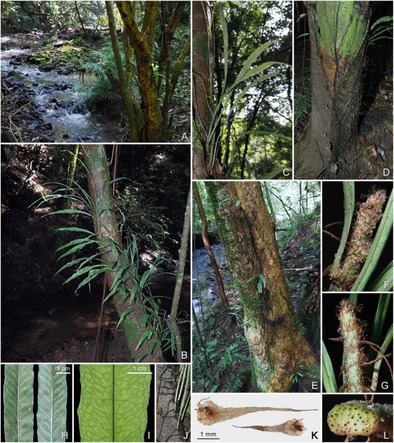

## 1 Introduction

The genus *Leptochilus* Kaulfuss (1824, 147), a member of the family Polypodiaceae, is primarily distributed across subtropical and tropical regions of Asia, Australia, as well as adjacent Pacific islands. This genus is morphologically characterized by long‐creeping rhizomes bearing clathrate to subclathrate scales that typically do not fully cover the rhizome; laminae ranging from simple to pinnatifid; anastomosing venation; and sori that are orbicular, elongate to linear, or occasionally acrostichoid. The genus *Leptochilus* displays extensive morphological variation, making it one of the most taxonomically challenging groups within Polypodiaceae. The taxonomic chaos has led to ongoing debate regarding the number of species within this genus. Based on a sampling of 40 *Leptochilus* species, Zhang et al. (2019), Zhang, Lian, et al. (2024), and Zhang, Lu, et al. (2024) conducted a comprehensive phylogenetic study and proposed that the genus comprises approximately 50 species. However, only 33 *Leptochilus* species were accepted by Govaerts et al. (2021).

China stands out as the epicenter of *Leptochilus* species globally, hosting 31 species (Zhang, Lian, et al. 2024), which include a newly published species designated as *L. tridigitatus* H.J.Wei (Wei et al. 2024). Interestingly, among these, only one species, *L. scandens* H.J.Wei & Yi Huang, is noted for its hemiepiphytic growth habit (Zotz et al. 2021; Wei et al. 2023). Herein, we introduce an additional hemiepiphytic *Leptochilus* species.

In 2022, the first author had photographed a plant belonging to the genus *Leptochilus* and named *Leptochilus leveillei* (Christ) X. C. Zhang & Noot. during a vascular plant survey in the Junzifeng National Nature Reserve in Fujian Province, southeastern China. The species was first observed as an epiphyte growing on *Adina pilulifera* (which belongs to the family Rubiaceae). However, one of the authors, Hong‐Jin Wei, learned that the plant was found climbing on some higher tree trunks, suggesting that the newly discovered plant might be a hemiepiphyte. This hypothesis was corroborated by the subsequent field investigations, which indicate that the plant should represent a different taxon. Morphologically, this plant is superficially similar to *L. leveillei* , an epilithic or terrestrial species that occasionally can extend its rhizomes onto lower tree trunks. We confirmed that this novel species does not match any previously known *Leptochilus* species after morphological and phylogenetic studies. Here, we describe it as a new species and used molecular markers to explore its phylogenetic position. Additionally, we reported the plastid genome of this new species and used it to reconstruct phylogenetic relationships of the genus *Leptochilus*.

## 2 Material and Methods

### 2.1 Habitat Investigation and Sampling

The species was discovered in a stream habitat at Shuikou Village, Gaiyang Town, within the Junzifeng National Nature Reserve, Mingxi County, Fujian Province, China. An extensive field research was conducted in 2023. As many as possible similar plants were searched for in the area where the original observation had been made. Their growth habits, especially their roots, were observed carefully and photographed. Several samples of the species were collected from different individuals. Some individuals were taken to the greenhouse in Shanghai Chenshan Botanical Garden for further study.

### 2.2 Ploidy Assessment

Fresh leaves of the individuals in the greenhouse introduced from the field were used to estimate the levels of ploidy by flow cytometry.

### 2.3 Morphological Description

Morphological data of the plants was obtained from the specimens collected during the fieldwork. Vouchers were deposited in the Dendrological Herbarium of Forestry College, Fujian Agriculture and Forestry University (FJFC) and Shanghai Chenshan Herbarium (CSH). The specimens as well as rhizome scales were photographed with a digital camera (OM SYSTEM OM‐1) and a stereo microscope (Nikon SMZ18), respectively. Comparative morphological analysis of the collected material and specimens of *Leptochilus henryi* (CSH0038826, CSH0038838, PE02051386) and *L. scandens* (CSH0193333) support its recognition as a distinct species within the genus *Leptochilus*. Fresh rhizomes were sectioned using a Flying Eagle brand single‐edged safety razor blade and subsequently examined under a microscope.

### 2.4 Taxon Sampling and Sequencing

Fresh leaves were dried in silica gel. Total DNA was extracted using the cetyltrimethylammonium bromide (CTAB) method (Allen et al. 2006) and subsequently sequenced on an Illumina HiSeq 4000 platform (Illumina, San Diego, CA, USA), yielding 150‐bp paired‐end reads. Over 30 Gb raw data were generated. Quality control was conducted by FastQC (http://www.bioinformatics.babraham.ac.uk/projects/fastqc/) with default parameters.

### 2.5 Plastome Assembly and Annotation

The complete plastid genome of this new species was assembled by the GetOrganelle pipeline (Jin et al. 2020). The Plastid Genome Annotator (PGA) software (Qu et al. 2019) was used to annotate the complete plastid genomes, and the published sequence of *Leptochilus decurrens* (MN044573) was used as a reference. The annotated plastomes were manually adjusted in Geneious R11.1.5 (Kearse et al. 2012). The annotation circle maps were drawn by OGDRAW (Greiner et al. 2019).

### 2.6 Phylogenetic Analyses

In this study, species circumscriptions follow the framework established by Zhang et al. (2019), Zhang, Lian, et al. (2024), and Zhang, Lu, et al. (2024). A total of 39 *rbcL*, 40 *rps4* and *rps4*‐*trnS*, and 38 *trnL* + *trnL‐trnF* sequences from 42 taxa were downloaded from GenBank to reconstruct the phylogenetic tree (Table 1). A total of 14 plastome sequences were downloaded from GenBank to reconstruct the phylogenomic relationship (Table 2). The molecular sequences were aligned using MAFFT (Katoh et al. 2002) with default parameters. The aligned sequences were then concatenated into a single matrix using the “Concatenate Sequence” script in PhyloSuite (Zhang et al. 2020). The phylogenetic trees were inferred using IQ‐TREE (Nguyen et al. 2014), incorporating the ultrafast bootstrap (UFBoot) and the approximate Bayes Test.

**TABLE 1:** Voucher and GenBank accession information of each species used. A dash (—) indicates missing data.

Scientific name	Voucher	*rbcL*	*trnL + trnL‐F*	*rps4* + *rps4‐trnS*
*Leptochilus bolikhamsaiensis* Liang Zhang, Khamphanh Thepkaysone & Zhuo Zhou	Zhuo Z. et al. LZ156 (KUN)	PQ316915	PQ317022	PQ316962
*Leptochilus cantoniensis* (Baker) Ching	Dong S.‐Y. 1034 (IBSC)	EU363245	—	EU363258
*Leptochilus* cf. *ellipticus*	Chen C.‐C. 1065 (H)	MH665038	MH665169	MH665102
*Leptochilus* cf. *flexilobus*	Zhang L.‐B. et al. 6710 (CDBI, MO, VNMN)	MH768417	MH768545	MH768479
*Leptochilus* cf. *hemitomus*	Zhang L.‐B. et al. 6484 (CDBI, MO, VNMN)	MH768427	MH768555	MH768489
*Leptochilus* cf. *pothifolius*	Cicuzza D. 1998 (HITBC)	PQ316873	PQ317004	PQ316943
*Leptochilus chingii* Li Bing Zhang & Liang Zhang	Zhang L.‐B. et al. 7453 (CDBI, MO, VNMN)	MH768437	MH768565	MH768502
*Leptochilus daklakensis* Liang Zhang, Li Bing Zhang, X.M.Zhou & Thien Tam Luong	Zhang L.‐B. et al. 8944 (CCDBI)	PQ316871	PQ317002	PQ316941
*Leptochilus digitatus* (Baker) Noot.	A.R. Smith 00–036 (UC)	EU482948	EU483044	EU482998
*Leptochilus dissimilialatum* (Bonap.) Li Bing Zhang & Liang Zhang	Zhang L.‐B. et al. 6362 (CDBI, MO, VNMN)	MH768419	MH768547	MH768481
***Leptochilus dolichophyllus* H.H.Fu & H.J.Wei**	She‐Lang Jin, Hou‐Hua Fu JSL9400	PV442128	PV442128	PV442128
*Leptochilus flexilobus* (Christ) Li Bing Zhang & Liang Zhang	Zhang L. et al. 3050 (KUN)	PQ316887	PQ317020	PQ316960
*Leptochilus gracilis* Z.L.Liang, Liang Zhang & Li Bing Zhang	Liang Z.‐L. et al. 607 (KUN, CDBI)	—	MW142228	MW142229
*Leptochilus hemionitideus* (C.Presl) Noot.	Wu S.‐K. et al. WS‐2437 (KUN)	JX103694	JX103778	JX103736
*Leptochilus hemitomus* (Hance) Noot.	Zhang X.‐C. 3302 (PE)	EU482951	EU483047	EU483001
*Leptochilus henryi* (Baker) X.C.Zhang	DJY04047 (CDBI)	MH768428	MH768556	MH768490
*Leptochilus heterophyllus* (S.K.Wu & K.L.Phan) Christenh.	Wu S.‐K. et al. WP‐136 (KUN)	JX520933	JX520937	JX520935
*Leptochilus kachinensis* Liang Zhang & Li Bing Zhang	Deng Y.‐F. et al.3200 (CDBI)	PQ316865	PQ316996	PQ316936
*Leptochilus khammouanensis* Liang Zhang, Thepkaysone & Z.Zhou	Shui Y.M. et al. LK136 (KUN)	PQ316872	PQ317003	PQ316942
*Leptochilus leveillei* (Christ) X.C.Zhang & Noot.	Zhang L.‐B. et al. 475 (CDBI)	PQ316867	PQ316998	PQ316938
*Leptochilus leveillei* (Christ) X.C.Zhang & Noot.	Zhang X.‐C. 4312 (PE)	MH665047	MH665179	MH665112
*Leptochilus leveillei* (Christ) X.C.Zhang & Noot.	Zhang L.‐B. et al. 6623 (CDBI)	—	PQ316990	PQ316929
*Leptochilus leveillei* (Christ) X.C.Zhang & Noot.	Zhang L.‐B. et al. 7069 (CDBI)	PQ316861	PQ316991	PQ316930
*Leptochilus luangprabangensis* Liang Zhang & Zhuo Zhou	Zhuo Z. et al. LZ415 (KUN)	PQ316898	PQ317034	PQ316974
*Leptochilus macrophyllus* (Blume) Noot.	Wade 1772 (TAIF)	MH768447	MH768573	MH768511
*Leptochilus morsei* (Ching) Fraser‐Jenk.	Jin S.‐L. et al. JSL7322 (CSH)	—	PQ317014	PQ316954
*Leptochilus multilobus* Liang Zhang & X.M.Zhou	YLZB2004 (CDBI)	PQ316868	PQ316999	—
*Leptochilus oblongus* Li Bing Zhang, Liang Zhang & N.T.Lu	Zhang L.‐B. et al. 6299 (CDBI, MO, VNMN)	MH768429	MH768557	MH768491
*Leptochilus ovatifolius* Zhe Zhang, S.W.Yao & Yi Huang	Zhang L. NAS20180929_4 (KUN)	PQ316880	—	PQ316951
*Leptochilus ovatus* Copel.	Wade 1583 (TAIF)	PQ316863	PQ316994	PQ316934
*Leptochilus pentaphyllus* (Baker) Li Bing Zhang & Liang Zhang	Xu C.‐D. A0357 (PE)	MH665043	MH665175	MH665108
*Leptochilus pteropus* (Blume) Fraser‐Jenk.	Zhang L.‐B. et al. 8014 (CDBI, MO, VNMN)	MH768412	MH768540	—
*Leptochilus sanjiangensis* Liang Zhang & Li Bing Zhang	Jin S.‐L. et al. JSL7568 (CSH)	PQ316889	PQ317025	PQ316965
*Leptochilus saxicola* (H.G.Zhou & H. Li) Li Bing Zhang & Liang Zhang	Zhang L.‐B. et al. 6772 (CDBI, MO, VNMN)	MH768441	MH768569	MH768505
*Leptochilus scandens* H.J.Wei & Yi Huang bis	Jin S.‐L. et al. JSL8000 (CSH)	PQ316911	—	PQ316985
*Leptochilus shintenensis* (Hayata) X.C.Zhang & Noot.	Knapp R. 3874 (P)	MH768454	—	MH768518
*Leptochilus* sp.	Knapp R. 3849 (P)	MH768443	MH768571	MH768507
*Leptochilus wrightii* (Hook. & Baker) X.C.Zhang	Li 47 (PE)	MH665064	MH665197	MH665130
*Leptochilus wusugongii* Liang Zhang & Li Bing Zhang	Wu S.‐K. et al. WS‐2591 (KUN)	PQ316874	PQ317005	PQ316944
Outgroup				
*Microsorum commutatum* Copel.	Wade 3768 (TAIF)	MH051171	MH113503	MH113470
*Microsorum insigne* (Blume) Copel.	Liu 204 (PE)	EU482957	EU483054	EU483008
*Microsorum punctatum* (L.) Copel.	Wade 1390 (TAIF)	MH051177	MH113509	MH113476
*Phymatosorus longissimus* (Blume) Pic.Serm.	Cheng X. et al. FB042 (KUN)	MT130640	MT130640	MT130640

**TABLE 2:** Scientific names and accessions information of the specimens used in this study.

Scientific name	Accession number
*Leptochilus decurrens* Blume	MN044573/MW876331
***Leptochilus dolichophyllus* H.H.Fu & H.J.Wei**	PV442128
*Leptochilus ellipticus* (Thunb.) Noot.	MW876332/MT130679
*Leptochilus hemionitideus* (C.Presl) Noot.	MH319943
*Leptochilus henryi* (Baker) X.C.Zhang	MW876333
*Leptochilus macrophyllus* (Blume) Noot.	MW876334
*Leptochilus pteropus* (Blume) Fraser‐Jenk.	MW876341
*Leptochilus* sp.	MZ019431
Outgroup	
*Microsorum insigne* (Blume) Copel.	MW876340
*Microsorum punctatum* (L.) Copel.	MW876342
*Microsorum steerei* (Harr.) Ching	MW876343
*Phymatodes longissimi* (Blume) J.Sm.	MT130640
*Phymatosorus cuspidatus* (D.Don) Pic.Serm.	MW876350

## 3 Results

### 3.1 Morphological Characters

Morphologically, a detailed comparison between the new species and its morphologically most similar species, *L. leveillei*, shows that the new species can be distinguished by narrower rhizome scales, shorter stipes, and longer lamina (Table 3).

**TABLE 3:** Morphological comparison between *Leptochilus dolichophyllus* and *Leptochilus leveillei* .

Character	*L. dolichophyllus*	*L. leveillei*	*L. scandens*
Rhizome scale shape	Narrowly lanceolate to narrowly ovate‐lanceolate	Ovate to ovate‐lanceolate	Lanceolate to ovate‐lanceolate
Rhizome scale color	Dark brown or nearly dark	Brown	Dark brown
Rhizome scale margin	Nearly entire or sparsely denticulate	Nearly entire or sparsely denticulate	Nearly entire, occasionally with 1 or 2 min teeth
Rhizome scale apex	Long acuminate	Acuminate	Acuminate
Frond dimorphism	Slightly dimorphic	Monomorphic or slightly dimorphic	Monomorphic
Fertile frond shape	Linear to linear lanceolate	Oblanceolate to linear lanceolate	Ovate to broadly ovate, deeply pinnatifid
Fertile lamina size	38.0–63.0 × 1.0–2.0 cm	20.0–40.0 × 1.0–4.0 cm	9.0–21.0 × 5.0–18.0 cm
Stipe length	0.4–3.0 cm	4.0–8.0 cm	5.0–28.0 cm
Life form	Hemiepiphytic	Terrestrial or epilithic	Hemiepiphytic

### 3.2 Characteristics of the Plastid Genome

**FIGURE 1:**
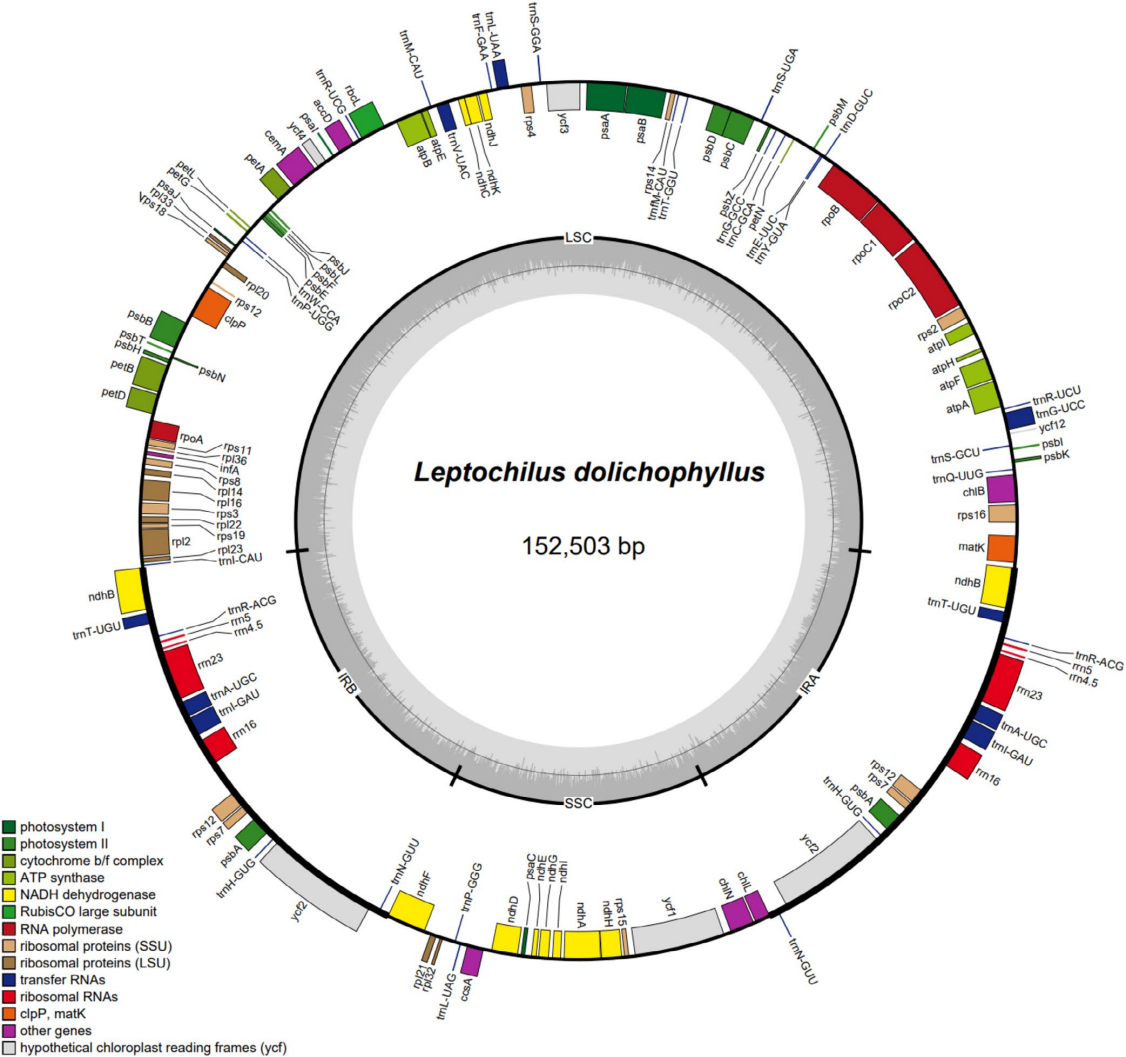
The plastome annotation map of *Leptochilus dolichophyllus*. The darker gray in the inner circle corresponds to GC content. The IRA and IRB (two inverted repeating regions); LSC (large single‐copy region); and SSC (small single‐copy region) are indicated outside of GC content.

We assembled the complete plastid genome of this new species, which possesses a total genome size of 152,503 bp with 44.0% GC content (Figure 1). The plastome displayed a typical quadripartite structure with a pair of IRs (24,631 bp), an LSC region (81,437 bp), and an SSC region (21,804 bp). The plastomes of this new species encoded 132 genes, comprising 89 protein‐coding genes, 35 transfer RNA (tRNA) genes, and 8 ribosomal RNA (rRNA) genes.

### 3.3 Characteristics of Life Form

Ecologically, all individuals of the plant at various growth stages are observed growing on tree trunks near a stream within a broad‐leaved forest. Most individuals are hemiepiphytic on *A. pilulifera*, and occasionally on *Alniphyllum fortunei* (which belongs to the family Styracaceae). They typically occur at heights above 1.5 m, with some reaching up to 2.0 m. The juvenile plants are initially epiphytic. The roots emerged from the rhizomes and extended toward the ground. Mature individuals consistently had established root contact with the soil, indicating a transition from an epiphytic to a hemiepiphytic stage. This demonstrates that the plant is unequivocally a hemiepiphyte and represents a distinct taxon from *L. leveillei* .

### 3.4 Characteristics of Ploidy and Genome Size

Cytologically, the plant showed a mean DNA content of 11.81 Gb, similar to that of *L. scandens* (11.82 Gb), and was inferred to be diploid.

### 3.5 Phylogenetic Relationships

The aligned matrix lengths for plastid regions *rbcL*, *rps4* + *rps4‐trnS*, and *trnL* + *trnL‐trnF* sequences were 1269, 1108, and 926 bp, respectively. The concatenated matrix length was 3303 bp, with 818 (24.77%) variable sites and 390 (11.81%) parsimony informative sites.

**FIGURE 2:**
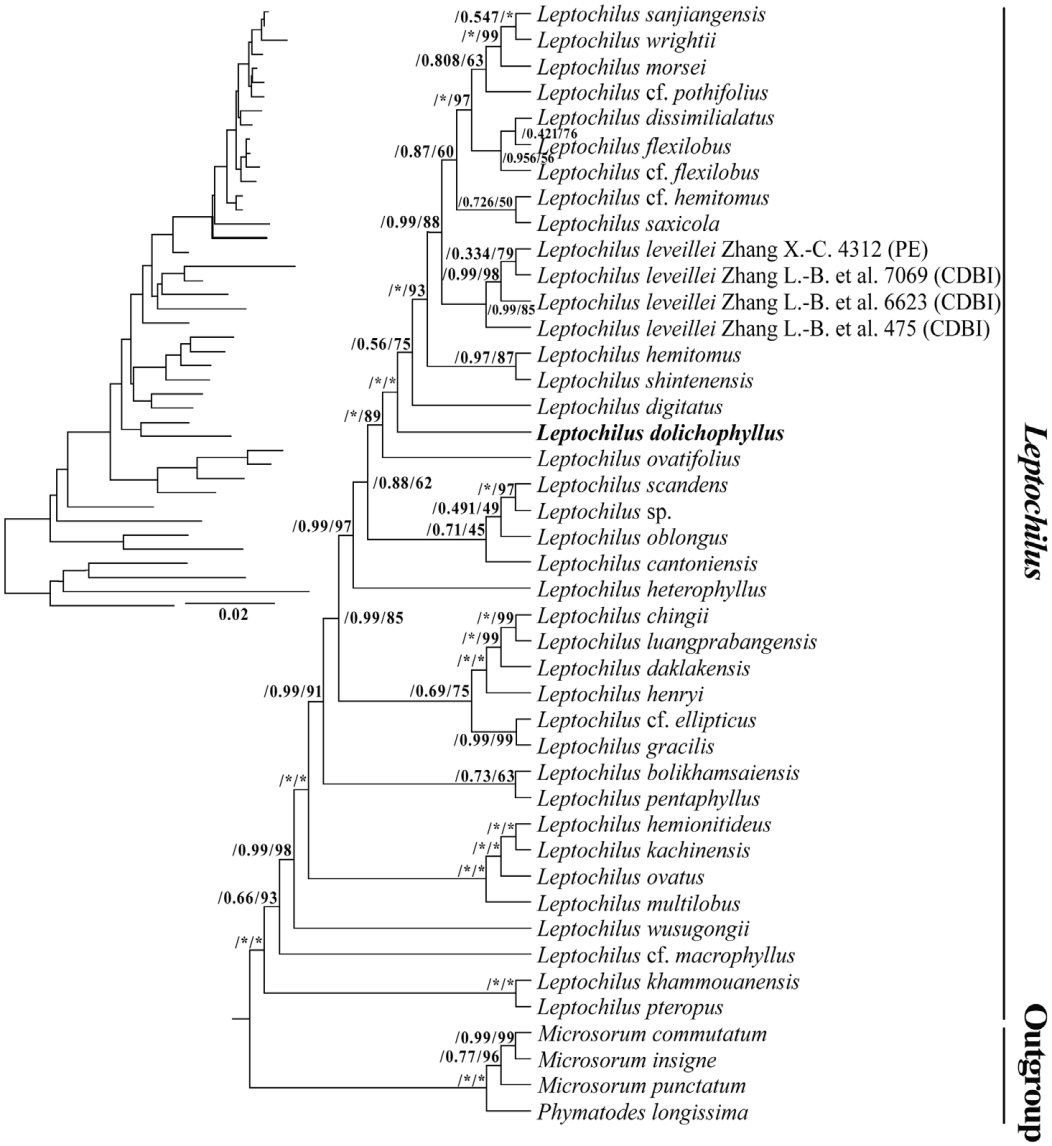
ML phylogenetic tree based on combined plastid genome regions (*rbcL*, *rps4* + *rps4‐trnS*, and *trnL* + *trnL‐trnF*). The numbers near the nodes are approximate Bayesian posterior probabilities and bootstrap percentages (aBayes supports and BP). *Node is the 1.00 posterior probability or 100 bootstrap percentage.

**FIGURE 3:**
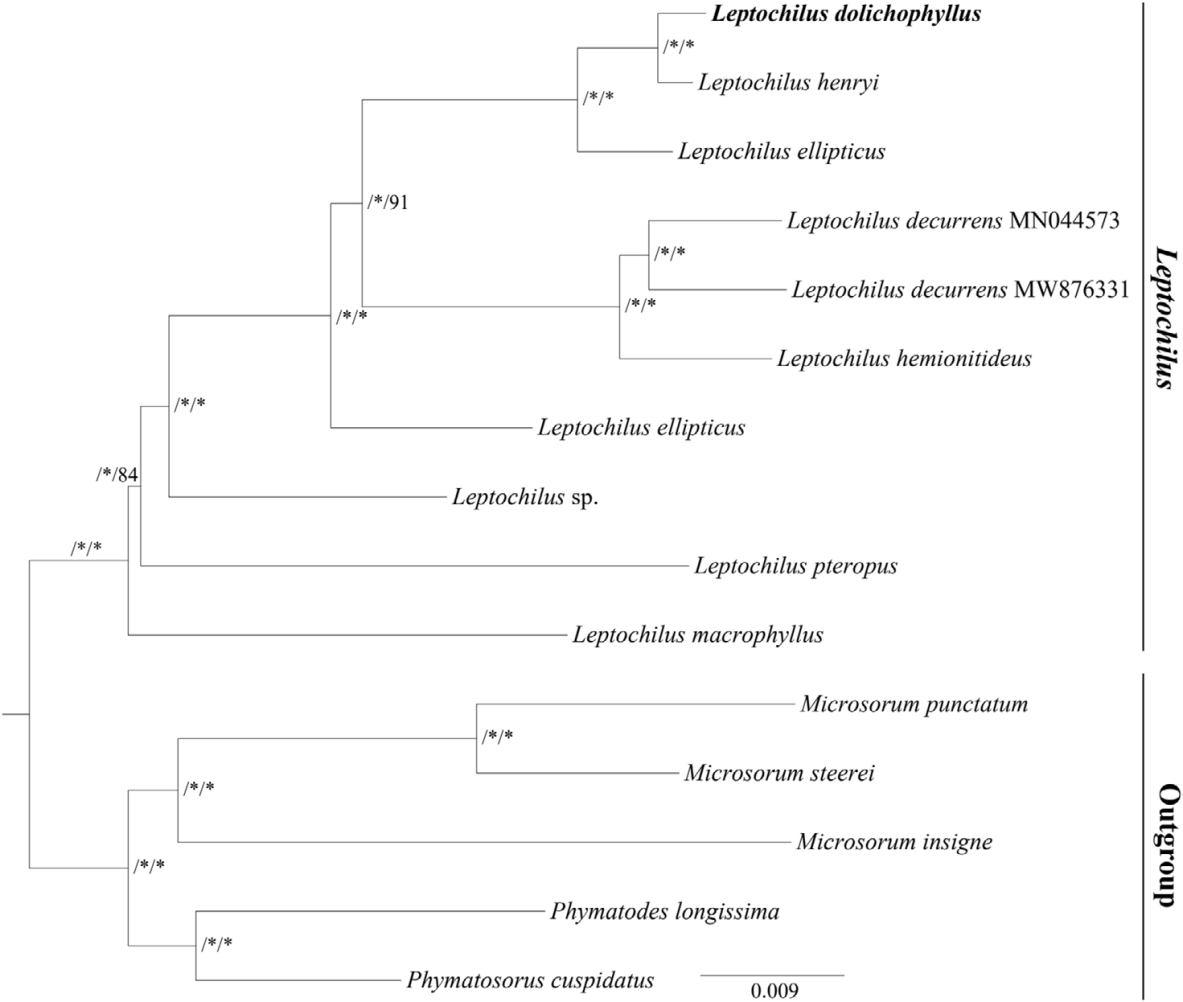
Phylogenetic tree obtained by ML analysis of the plastomes matrix. The numbers near the nodes are approximate Bayesian posterior probabilities and bootstrap percentages (aBayes supports and BP). *Node is the 1.00 posterior probability or 100 bootstrap percentage.

The phylogenetic relationships of the genus *Leptochilus* were reconstructed based on three traditional molecular markers, indicating that the new *Leptochilus* species formed a distinct clade with strong support (Figure 2). Additionally, we conducted a phylogenetic analysis based on complete plastome sequences, indicating that the *Leptochilus* species formed a well‐supported monophyletic group (aBayes = 1.00, BS = 100) (Figure 3). The new species was sister to *L. henryi* with strong support (aBayes = 1.00, BS = 100).

## 4 Discussion

This new species is the ninth species of the genus *Leptochilus* discovered in Fujian Province (An et al. 2023). Additionally, this species represents the second known case of a hemiepiphytic growth habit within the genus in China, with *L. scandens* previously being the only confirmed species exhibiting such a habit (Wei et al. 2023). Within the genus *Leptochilus*, many species are epilithic, epiphytic, or terrestrial, with a few capable of climbing onto lower trunks of trees. Although certain terrestrially germinating species like *L. mengsongensis* M.X.Zhao and *L. axillaris* (Cav.) Kaulf. demonstrate climbing capabilities reaching up to 2 m on tree trunks, they are not classified as hemiepiphytes under the criteria established by Zotz et al. (2021). Zhang, Lu, et al. (2024) recently reported another simple‐fronded hemiepiphyte, *L. luangprabangensis* Liang Zhang, Khamphanh Thepkaysone & Zhuo Zhou from Vietnam; however, the study lacks in situ photographic documentation to substantiate this claim, such as images of epiphytic juvenile plants and subadult or adult plants with adventitious roots anchoring to the ground, as those shown by Fujiwara et al. (2023) and Wei et al. (2023).

Previous phylogenetics studies of *Leptochilus* were mainly inferred by traditional molecular markers (Schneider 2004; Dong et al. 2008; Kreier et al. 2008; Kim et al. 2012; Testo and Sundue 2014). However, the interspecific relationships are still unclear, with moderate to weak support at numerous nodes, indicating that the phylogenetic relationships within the genus remain unresolved. Our phylogenetic analyses were consistent with previous results, and the support of clades was low to moderate (Figure 2). Further studies with broader sampling and molecular markers containing more loci are needed.

Due to the relatively simple and conserved structure, abundant informative sites, and well‐understood genetic background, plastomes have become an effective molecular resource for resolving complex phylogenetic relationships (Gitzendanner et al. 2018). However, plastome‐based studies of the genus *Leptochilus* have mostly focused on subfamily‐level phylogenies (Liu et al. 2021; Wei et al. 2021) or have been limited to reports of individual species' plastome sequences (Min et al. 2018; Su et al. 2019). To date, only nine plastid genomes of *Leptochilus* have been deposited in GenBank, most of which lack annotation information, hindering our understanding of the evolutionary history and phylogenetic relationships within the genus. To provide reliable molecular data for future phylogenetic studies of the genus *Leptochilus*, we assembled and annotated the plastome of the new species. The results revealed that its plastome has a genome size of 152,503 bp and contains 132 genes, which is entirely within the previously reported size range of *Leptochilus* plastomes, from 152,084 bp ( *L. macrophyllus* ) to 157,508 bp (*L. pteropus*) (Min et al. 2018; Su et al. 2019). The phylogenetic analysis based on whole plastome sequences showed that species of this genus form a highly supported monophyletic group, and the interspecific phylogenetic resolution has greatly improved. The newly described species is sister to *L. henryi* (aBayes = 1.00, BS = 100). This result indicates that plastome sequences are well suited for resolving intrageneric relationships within *Leptochilus*.

Overall, integrative evidence from ecological niche differentiation, morphometric disparity, and molecular phylogenetic divergence robustly corroborates the recognition of this species as a distinct species from *L. leveillei* .

## 5 Taxonomy Treatment

**FIGURE 4:**
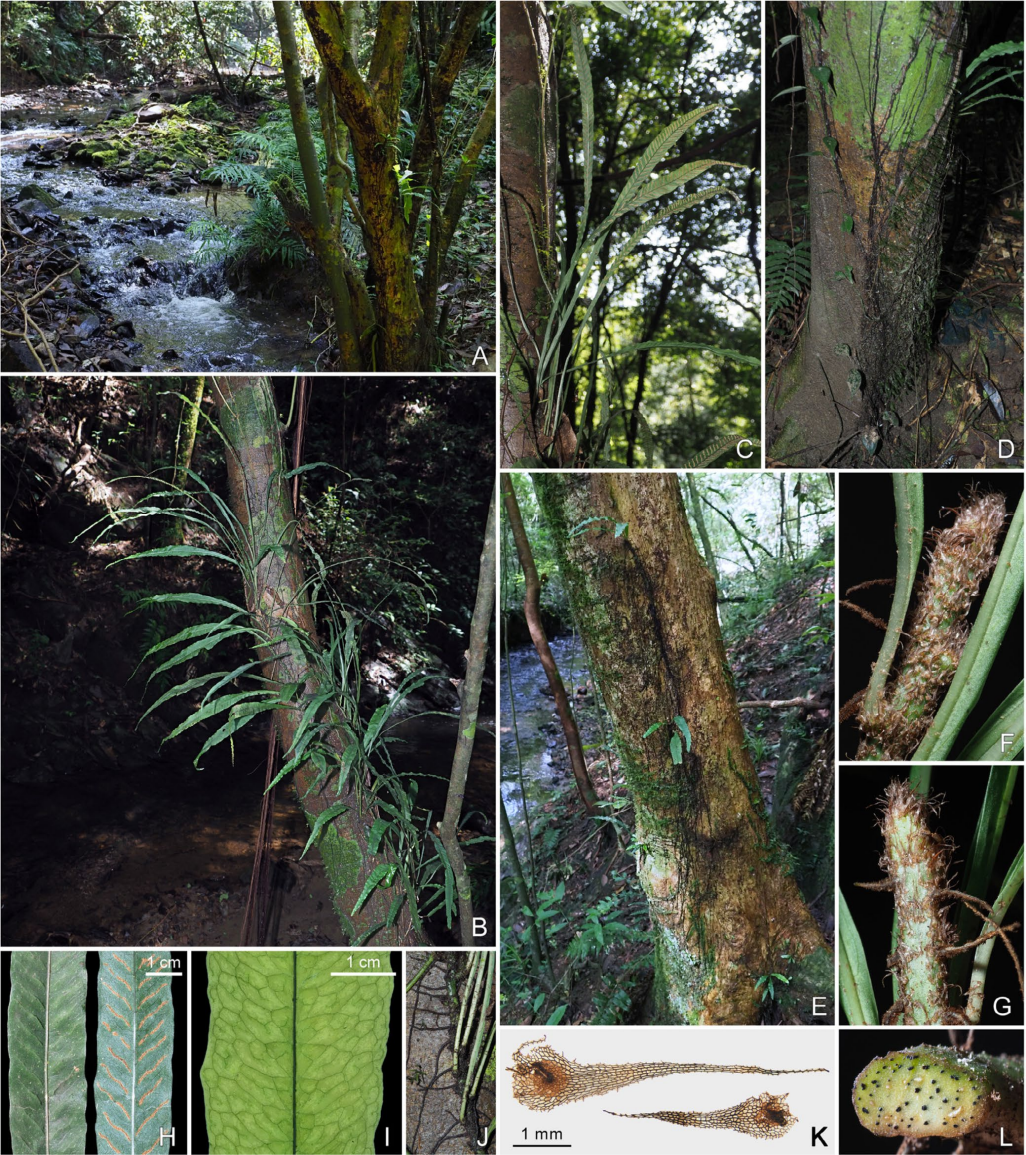
Habitat and morphology of *Leptochilus dolichophyllus*. (A) Habitat. (B) Habit. (C) Fertile fronds. (D) Adventitious roots establishing terrestrial connection. (E) Epiphytic young individuals with roots extending downwards. (F) Dorsal view of rhizome. (G) Ventral view of rhizome. (H) Portion of fertile frond. (I) Portion of sterile frond showing venation. (J) Lower portion of stipes with rhizome. (K) Scales from rhizome. (L) Cross section of rhizome.

**FIGURE 5:**
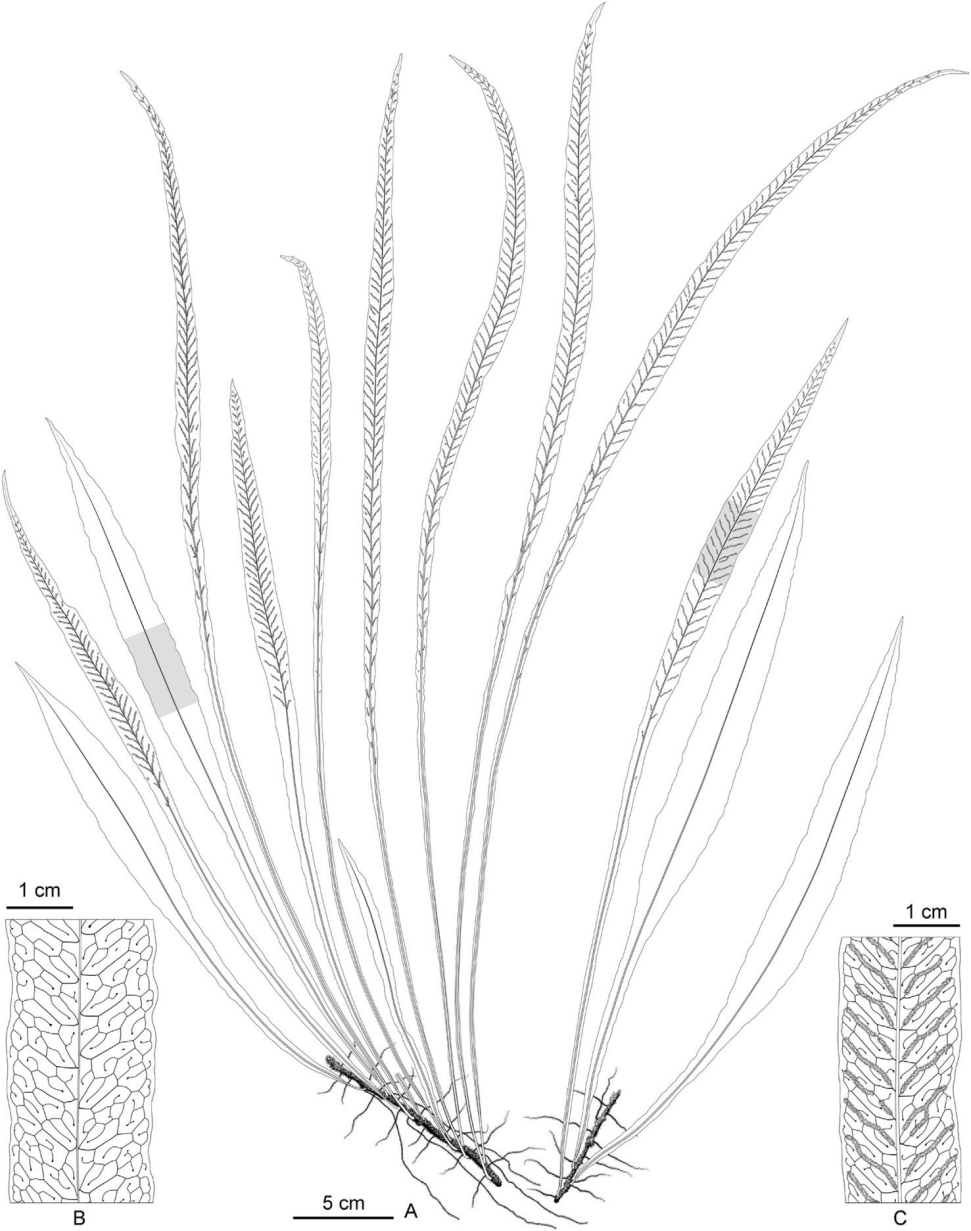
Line drawings of *Leptochilus dolichophyllus*. (A) Habit. (B) Portion of sterile frond showing venation. (C) Portion of fertile frond showing sori.

***Leptochilus dolichophyllus*** H.H.Fu & H.J.Wei, sp. nov. (Figures 4 and 5)

Type: CHINA. Fujian: Sanming, Jun Zi Feng National Nature Reserve, under evergreen broad‐leaved forest, on tree trunks near a stream, 26°32′51″ N, 117°3′37″ E, elev. 352 m, 18 Sep. 2023, *She‐Lang Jin, Hou‐Hua Fu JSL9400* (holotype: FJFC!, isotypes: CSH!, IBK!, KUN!, PE!).

### 5.1 Diagnosis

*Leptochilus dolichophyllus* is similar to *L. leveillei* , but differs by the narrower rhizome scales (narrowly ovate‐lanceolate, 2.0–6.0 mm vs. ovate‐lanceolate, 1.1–4.4 mm), shorter stipes (0.4–3.0 cm vs. 4.0–8.0 cm), longer fertile fronds (40.0–63.0 cm vs. 20.0–40.0 cm), and life form (hemiepiphytic vs. terrestrial or epilithic). Moreover, it can be distinguished from *L. scandens* by stipe length (0.4–3.0 cm vs. 5.0–28.0 cm), frond dimorphism (slightly dimorphic vs. monomorphic), and fronds (simple vs. pinnatipartite).

### 5.2 Description

Plants perennial, evergreen, hemiepiphytic. Rhizome slender, 2.0–3.0 mm in diam., dorsiventral, long scandent, scaly at apex; vascular bundles up to 10; scales pseudo‐peltate, dark brown or near dark, narrowly lanceolate to narrowly ovate‐lanceolate, 2.0–6.0 mm, margins nearly entire, or slightly denticulate, apex long acuminate. Fronds distant, slightly dimorphic, fertile fronds longer and narrower, stipe stramineous, much shorter than lamina, glabrous; venation visible, lateral veins straight or zigzag more or less and dichotomous distally, veinlets anadromous, thinner than lateral veins, included veinlets simple or forked; lamina simple, widest at middle, gradually decurrent nearly to stipe base, margin slightly undulate, herbaceous, glabrous on both surfaces, dark green adaxially, pale green abaxially. Sterile fronds: 27.0–42.0 cm long; stipe 3.0–10.0 mm long, 0.5–1.0 mm in diam. at middle; lamina lanceolate to narrowly lanceolate, 32.0–41.0 × 2.2–3.2 cm, apex acuminate to shortly acuminate; veins forming 2 or 3 rows of areoles between midrib and lamina margin. Fertile fronds: (38.0–)45.0–63.0 cm long, stipe 4.0–27.0 mm, 0.5–1.3 mm in diam. at middle, lamina linear to linear lanceolate, (37.0–)43.0–60.0 × 0.9–2.0 cm, apex long acuminate or caudate; veins forming 3 or 4 rows of irregularly arranged areoles between midrib and lamina margins, midrib raised on both surfaces. Sori linear, 1 regular row between lateral veins, from midrib or near midrib to near margin of lamina, at an angle of 30°–45° with midrib; paraphyses absent. Spores yellow, 64 per sporangium.

### 5.3 Geographical Distribution

**FIGURE 6:**
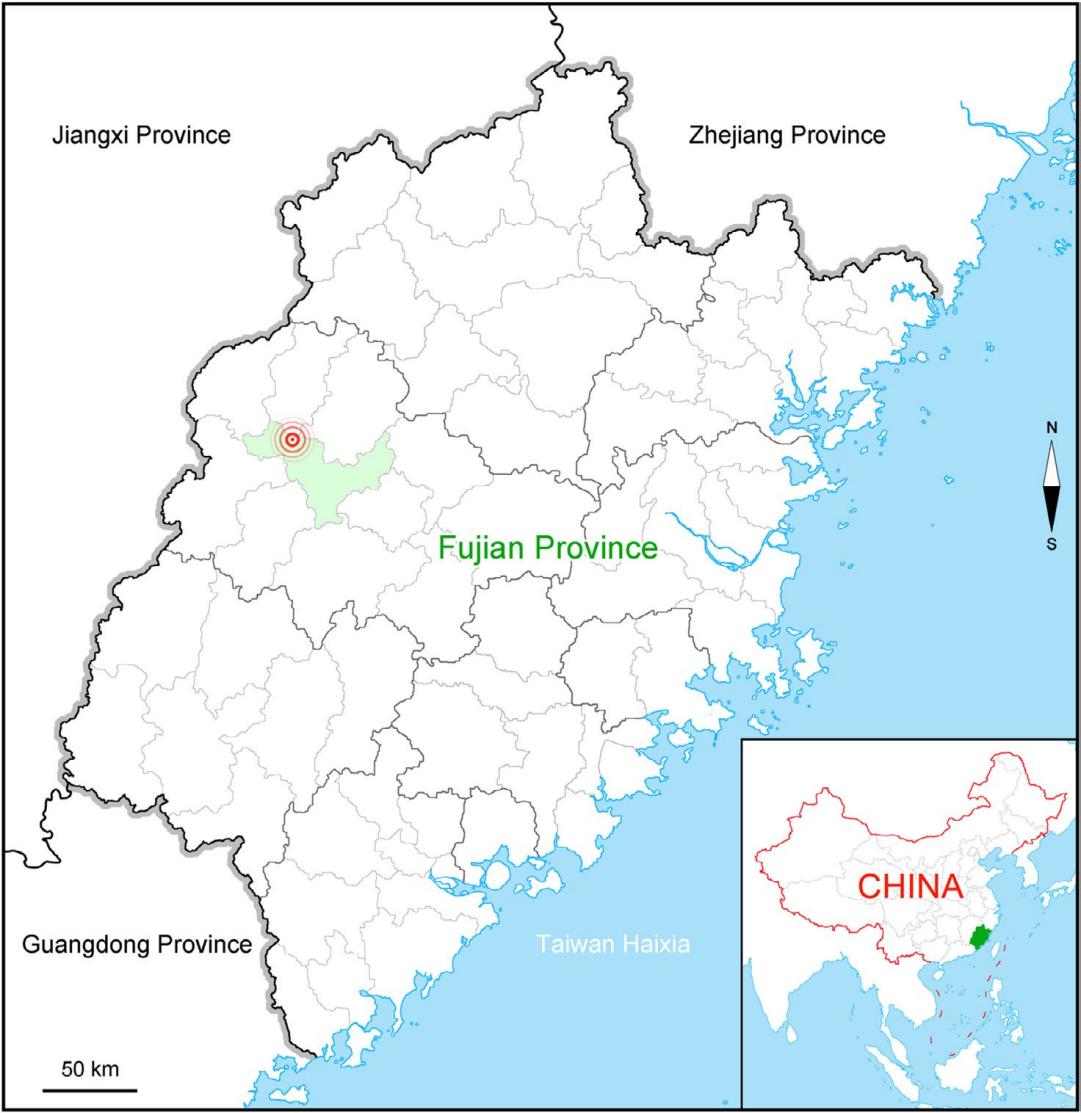
Geographical distribution of *Leptochilus dolichophyllus* (center of rings).

China, Fujian, Sanming, Junzifeng National Nature Reserve in Fujian (Figure 6).

### 5.4 Ecology

*Leptochilus dolichophyllus* mostly climbs on tree trunks in the understory of moist montane forest, about 350 m above mean sea level.

### 5.5 Etymology

The epithet of *L. dolichophyllus* is taken from the Greek compound word formed by “dolichos” (long) and “phyllon” (leaf), together meaning “long‐leaved,” referring to the elongated leaves morphology of the new species. Most individuals of *L. dolichophyllus* are hemiepiphytic on *A. pilulifera* and occasionally occur on *Al. fortunei*.

### 5.6 Vernacular Name

Simplified Chinese: 长叶线蕨; Chinese pinyin: cháng yè xiàn jué.

### 5.7 Conservation Assessments

The population of *L. dolichophyllus* covers an area of about 50–100 square meters, with about 50 individuals (clones of the same individual excluded) encountered in the field. As the species is currently known only from a single locality, we recommend its classification as Data Deficient under the IUCN Red List criteria (IUCN 2012).

## Author Contributions

**Hou‐Hua Fu:** conceptualization (equal), data curation (equal), formal analysis (lead), investigation (equal), visualization (equal), writing – original draft (equal). **Cheng‐Yuan Zhou:** data curation (equal), software (lead), visualization (supporting). **Xiong‐De Tu:** data curation (equal), validation (equal). **Liang Ma:** conceptualization (equal), investigation (equal), methodology (equal). **Shi‐Pin Chen:** conceptualization (equal), funding acquisition (lead), methodology (equal), project administration (lead), writing – review and editing (equal). **Hong‐Jin Wei:** investigation (equal), visualization (lead), writing – original draft (equal), writing – review and editing (equal).

## Conflicts of Interest

The authors declare no conflicts of interest.

## Data Availability

The DNA sequences generated in this study have been deposited in the National Center for Biotechnology Information (NCBI) database. The accession numbers and the information on the voucher specimens are available in Tables 1 and 2.
